# Combining the Suicide Intent Scale and the Karolinska Interpersonal Violence Scale in suicide risk assessments

**DOI:** 10.1186/s12888-015-0607-6

**Published:** 2015-09-23

**Authors:** J. Stefansson, P. Nordström, B. Runeson, M. Åsberg, J. Jokinen

**Affiliations:** Department of Clinical Neuroscience, Psychiatry Section, Karolinska Institutet, Karolinska University Hospital, Building R5, Solna, SE-171 76 Stockholm Sweden; Department of Clinical Sciences, Karolinska Institutet, Danderyd Hospital, Stockholm, Sweden; Department of Clinical Sciences, Umeå University, Umeå, Sweden

**Keywords:** Suicide, Suicide attempt, Risk factors, Aggression, Psychometrics, Violence

## Abstract

**Background:**

High suicide intent, childhood trauma, and violent behavior are risk factors for suicide in suicide attempters. The aim of this study was to investigate whether the combined assessment of suicide intent and interpersonal violence would provide a better prediction of suicide risk than an assessment of only suicide intent or interpersonal violence.

**Methods:**

This is a cohort study involving 81 suicide attempters included in the study between 1993 and 1998. Patients were assessed with both the Suicide Intent Scale (SIS) and the Karolinska Interpersonal Violence Scale (KIVS). Through the unique personal identification number in Sweden, patients were linked to the Cause of Death Register maintained by the Swedish National Board of Health and Welfare. Suicides were ascertained from the death certificates.

**Results:**

Seven of 14 patients who had died before April 2013 had committed suicide. The positive predictive value for the Suicide Intent Scale alone was 16.7 %, with a specificity of 52 % and an area under the curve of 0.74. A combined assessment with the KIVS gave higher specificity (63 %) and a positive predictive value of 18.8 % with an AUC of 0.83. Combined use of SIS and KIVS expressed interpersonal violence as an adult subscale gave a sensitivity of 83.3 %, a specificity of 80.3 %, and a positive predictive value of 26 % with an AUC of 0.85. The correlation between KIVS and SIS scores was not significant.

**Conclusions:**

Using both the the SIS and the KIVS combined may be better for predicting completed suicide than using them separately. The nonsignificant correlation between the scales indicates that they measure different components of suicide risk.

**Electronic supplementary material:**

The online version of this article (doi:10.1186/s12888-015-0607-6) contains supplementary material, which is available to authorized users.

## Background

A history of attempted suicide is the strongest known clinical risk factor for suicide. The suicide risk assessment is an important and difficult part of the management of patients after a suicide attempt. Among hospitalized male suicide attempters with bipolar/unipolar disorder and schizophrenia, almost 25 % committed suicide within the year following the suicide attempt [1]. Furthermore, suicide attempters who had used a violent method showed a very high risk [2].

There is insufficient evidence concerning the predictive value of structured suicide risk assessment scales. Clinical guidelines such as NICE, recommend, when assessing the risk of repetition of suicidal behavior, identification of the specific risks for the patient, taking into account methods and frequency of current and past suicidal behavior, current and past suicidal intent, depressive symptoms or any psychiatric illness and their relationship to self-harm, as well as the personal and social context and any other specific factors preceding self-harm [3]. They do not recommend the use of risk assessment tools and scales to predict future suicide or repetition of self-harm due to a lack of research evidence [3]. They mention in an authors’ reply regarding the NICE guidelines that the ‘positive predictive value’ of scales that examined the risk of suicide following self-harm ranged from 1 to 13 % [4]. This means that 87 to 99 % of those rated as being at high risk in these studies did not go on to die by suicide, thereby giving a low specificity in the prediction of suicide in self-harm patients [4]. Complicating the assessment strategy even further is the fact that most studies focus on single risk factors, leaving clinicians and expert panels to estimate how risk factors interact to influence outcomes [4]. In a recent review of practice in suicidal risk assessments, Fowler stressed the importance of using risk assessment scales with a multidimensional approach [5]. New standardized measures for the assessment of suicidal behavior and ideation have been designed to improve identification and clinical management and have recently been effectively disseminated, which may affect recommendations in future guidelines [6].

A frequently studied instrument for assessing suicide intent is the Beck Suicide Intent Scale (SIS) [7]. In a review article, five out of 13 studies showed a positive relationship between high SIS scores and suicide with follow-up periods ranging from 10 months to 20 years [8]. We have recently reported that high suicide intent, as well as high scores on the Planning subscale assessed shortly after a suicide attempt, predicted subsequent suicide [9].

Childhood trauma, adult violent behavior, and suicidal behavior are all interlinked [10, 11]. We have reported that high scores on exposure to violence as a child and expressed violent behavior as an adult, measured with the Karolinska Interpersonal Violence Scale (KIVS), predicted suicide in suicide attempters [12].

Earlier studies on structured suicide risk assessment scales for predicting suicide have pointed out the need for both a high sensitivity to detect the patients at risk for suicide and a better specificity to reduce the number of false positives. A combined or sequential screening method using largely orthogonal or independent variables that address both traits and state-dependent risk would be optimal for predicting suicide risk [13]. To the best of our knowledge, no studies have compared the combined use of two structured clinical instruments with the use of only one clinical rating scale in assessing suicide risk.

### Aims of the study

We hypothesized that combining two structured suicide risk assessment scales with some evidence of predictive ability for completed suicide, i.e., the Suicide Intent Scale and the Karolinska Interpersonal Violence Scale, would provide a better predictive value for case detection than using only one of the scales. Furthermore, we hypothesized that the scales measure different aspects of risk.

## Method

### Study setting

Patients being treated after a suicide attempt at the Karolinska University Hospital were asked to take part in a study on biological and psychological risk factors for suicidal behavior. The Regional Ethical Review Board in Stockholm approved the study protocol (Dnr 93–211) and the written informed consent of the participants was obtained.

### Patients

Between 1993 and 1998, 81 suicide attempters (35 men and 46 women, mean age 37 years, SD = 12, range 18–69 years) were included in the study from emergency departments and inpatient wards. Inclusion criteria were: a suicide attempt within 1 month of the evaluation, a minimum age limit of 18 years and ability to communicate both verbally and in writing in Swedish. Exclusion criteria were: schizophrenia spectrum psychosis, dementia, mental retardation, and intravenous drug abuse. This cohort is a part of the larger study reporting on the impact of a family history of suicide and childhood adversity on suicide risk [14]. As explained in our previous article [9], suicide attempt was defined as any nonfatal, self-injurious behavior with some intent to cause a completed suicide. A trained psychiatrist interviewed the participants using the SCID I research version interview to establish a diagnosis according to DSM-III. The SCID II interview was used to establish Axis II diagnoses. All clinical assessments and ratings were performed within 1 month of the suicide attempt.

The majority or, more precisely, 94 % of the suicide attempters had at least one current Axis I psychiatric diagnosis; 80 % satisfied the criteria for having a mood disorder, 5 % for an adjustment disorder, and 4 % for anxiety disorders; one patient had a substance-related disorder, one had anorexia nervosa, and one an unspecified psychiatric disorder (not psychotic) as the main Axis I diagnosis. The most usual comorbid substance-related disorder was alcohol dependence; 21 % of the suicide attempters had a substance-related disorder. Thirty-two (39.5 %) of the suicide attempters satisfied the criteria for a personality disorder; eight of them had a borderline personality disorder. Fourteen patients (17 %) had used a violent suicide attempt method. Forty-six patients (57 %) had a history of suicide attempts, and 19 of them (23 %) had attempted suicide three or more times before inclusion in the study.

### SIS and KIVS ratings

Beck’s Suicide Intent Scale (SIS) is a risk assessment instrument using 15-items designed to examine both subjective and objective aspects of the suicide attempt, such as the circumstances at the time of the attempt and the patient’s thoughts and feelings during the attempt [7]. We used item 18 of the SIS to categorize earlier suicide attempts: (1) none, (2) one or two, and (3) three or more earlier suicide attempts.

One patient’s SIS rating was incomplete and therefore was not used in the statistical analysis.

The Karolinska Interpersonal Violence Scale (KIVS) contains four rating scales assessing expressed violent behavior and exposure to violence in childhood (defined as 6–14 years of age) and during adult life (defined as age 15 or older) [12]. The ratings are based on a structured interview with concrete examples of interpersonal violence. The interrater reliability of the KIVS (and the subscales) was high and the scale has been validated with other clinical rating scales measuring aggression in suicide attempters [12]. Interviews and ratings (0–5 for each scale, total 20) were performed and assessed by trained clinicians. Two patients were not assessed by the KIVS.

### Assessment of mortality

All patients were followed up for mortality and cause of death. All deaths that occurred between enrollment in the study and April, 2013, were included. The patients who died during the follow-up period were identified and the causes of death were obtained from Statistics Sweden, which keeps the National Swedish Cause of Death Register for the Swedish National Board of Health and Welfare (http://www.socialstyrelsen.se). Seven suicides were ascertained from the death certificates.

### Data analysis

The population was characterized using the mean, median, and range for quantitative variables. The Shapiro-Wilks test was used to test whether the data were normally distributed. Parametric statistics (t test, one-tailed) was used for between-group comparisons, suicide victims vs. survivors, or first attempters vs. repeaters if the data were distributed normally. If the data were skewed, a nonparametric Wilcoxon test in continuous variables was applied for comparisons between groups. Fisher’s exact test (2-sided) was used in certain SIS and KIVS comparisons. Similarly to our previous SIS study [9], an *ad hoc* receiver-operating characteristic (ROC) analysis was used to find optimal thresholds for SIS and KIVS ratings to predict suicide. ROC tables and curves were created to establish the optimal cut-off values for completed suicide for both scales. ROC areas under the curves (AUCs) were determined as a measure of the diagnostic execution. They were then evaluated according to the methods of Hanley and McNeil. The chosen cut-off point for each scale was the one that both optimized the proportion of suicide victims correctly identified and the proportion of survivors correctly identified. We defined a positive result of the combined test (equivalent to high suicide risk) as when both instruments had scores over the thresholds established from ROC curves. A standard logistic regression analysis was performed using SIS and KIVS scores as predictors of suicide. Tests of non-parametric or parametric correlations were performed using Spearman’s rho or Pearson’s *r*. Statistical analyses were performed using the JMP 9.0.3 software from SAS Institute Inc., Cary, NC, USA. Confidence intervals for the AUCs were calculated with the SPSS statistical software package (IBM, SPSS^TM^, version 22). This article follows STROBE guidelines (see STROBE Checklist under Additional file 1).

## Results

### Suicides

Fourteen patients had died during the follow-up. Seven suicides (8.6 %), by three women and four men, were ascertained from the death certificates. Five suicide victims fulfilled the criteria for major depression, one for adjustment disorder, one suicide victim did not fulfill the criteria for an Axis 1 diagnosis at the inclusion to the study. Two of seven suicide completers had a comorbid alcohol abuse diagnosis and two had a comorbid personality disorder NOS diagnosis. There was no age difference between suicide victims and survivors. Five patients committed suicide within 6 years and two patients had died of suicide 11 years after entering the study (time to suicide: median 4 years, mean 6 years, range 1.7–12.8 years). The follow-up period ranged between 15 and 20 years. The proportion with suicide as the cause of death in the cohort was 50 % (7/14).

**Fig. 1:**
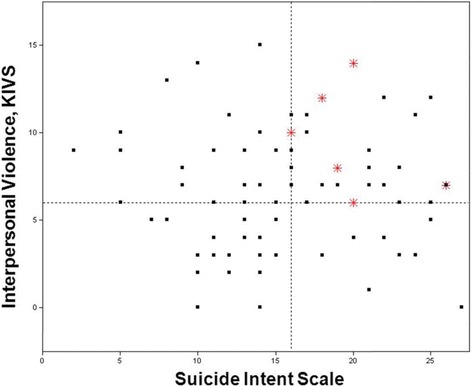
Correlation between SIS and KIVS total scores (*r* = −0.04, *p* = 0.74). * = completed suicides, dotted lines = cut-offs from ROC, SIS cut-off = 16. KIVS cut-off = 6

Table 1 shows the mean and median SIS and KIVS ratings for suicide victims and survivors. As reported earlier, both SIS and KIVS total scores were significantly higher in suicide victims than in survivors. One of the suicide victims had not been assessed by KIVS. Correlations between KIVS and SIS and the SIS planning subscale were not significant (Table 2). Figure 1 shows SIS and KIVS total scores for surviving suicide attempters and suicide victims.

**Table 1:** The ratings of suicide intent and interpersonal violence in suicide victims and survivors

	Suicide victims				Survivors				
Rating	Mean	Median	SD	Range	Mean	Median	SD	Range	Statistic
SIS	20.1	20	3.2	16–26	15.7	15	5.8	0–27	*t* ratio = 2.0 *p* = 0.026
SIS planning	10.1	10	2.5	7–15	7.3	7	3.8	0–15	*Z* = 2.0 *p* < 0.045
KIVS total	9.5	9	3.1	6–14	6.5	6	3.4	0–15	*t* ratio = 2.1 *p* = 0.018
Expressed violent behavior during childhood	1	1	0.9	0–2	0.8	1	0.8	0–5	*Z* = 0.8 *p* < 0.44
Expressed violent behavior as adult	2.5	2.5	1.0	0–4	1.4	2	1.1	0–5	*Z* = 2.3 *p* < 0.023
Exposure to violence during childhood	3.2	3.5	1.0	2–4	2.2	2	1.3	0–5	*Z* = 1.8 *p* < 0.073
Exposure to violence as an adult	2.8	3	1.0	1–4	2.1	2	1.4	0–5	*Z* = 1.3 *p* < 0.19

**Table 2:** The correlations (Spearman’s rho or Pearson’s r) between KIVS subscales and SIS and the SIS planning subscale

	Expressed violent behavior during childhood	Exposure to violence during childhood	Expressed violent behavior as an adult	Exposure to violence as an adult	KIVS total
SIS	−0.08	0.08	0.01	−0.04	*−0.04*
SIS planning	−0.06	−0.01	−0.13	−0.14	−0.11

### Repeated suicide attempts

Suicide attempters who reported earlier suicide attempts had significantly higher KIVS total scores compared with suicide attempters debuting with suicidal behavior at inclusion in the study (*Z* = −2.8, *p* = 0.0044). Repeaters scored higher on three out of four KIVS subscales: Exposure to interpersonal violence as a child (*Z* = −2.1, *p* = 0.034) and as an adult (*Z* = −2.6, *p* = 0.011), as well as Expressed violence as an adult (*Z* = −2.4, *p* = 0.014). There were no significant differences in SIS scores between repeaters and nonrepeaters (*p* = 0.86). There was no significant difference in suicide risk between repeaters and nonrepeaters (*p* = 0.98)

### Receiver-operating characteristic analysis

To estimate which cut-off level of SIS and KIVS scores optimally predicts suicide, we analyzed the ROC curves and tables. A SIS cut-off of 16 gave a sensitivity of 100 % and a specificity of 52 %. The positive predictive value (PPV) for SIS alone was 16.7 %, with an area under the curve (AUC) of 0.74. The KIVS total score cut-off of 6 gave a sensitivity of 100 % and a specificity of 40 %. The PPV for KIVS alone was 12.2 %, with an AUC of 0.75.

A combined assessment of both scales gave a sensitivity of 100 % and a specificity of 63 %. The PPV after combining both scales was 18.8 % with an AUC of 0.83 (Table 3).

**Table 3:** The positive predictive value and area under the curve when combining KIVS and SIS

Test	Cut-off	Suicide +	Suicide −	Sensitivity	Specificity	Positive predictive value	AUC 95 % CI *p* value
SIS	>16	7	35	100 %	52 %	16.7 %	0.74
*N* = 80				(7/7)	(38/73)	(7/42)	0.62–0.86
							0.037
	<16	0	38				
KIVS	>6	6	43	100 %	40 %	12.2 %	0.75
*N* = 78				(6/6)	(29/72)	(6/49)	0.58–0.92
							0.043
	<6	0	29				
SIS + KIVS		6	26	100 %	63 %	18.8 %	0.83
				(6/6)	(45/71)	(6/32)	0.70–0.95
							0.017
Combined *N* = 77		0	45				

Table 4 shows suicide risk assessments on combining SIS and two predictive KIVS subscales: Expressed interpersonal violence as an adult and Exposure to interpersonal violence as a child. Combined use of SIS and KIVS expressed interpersonal violence as an adult gave a sensitivity of 83.3 %, a specificity of 80.3 %, and a higher positive predictive value of 26 % with an AUC of 0.85.

**Table 4:** Positive predictive value and area under the curve on combining SIS and KIVS subscales

Test	Cut-off	Suicide +	Suicide −	Sensitivity	Specificity	Positive predictive value	AUC 95 % CI *p* value
KIVS violence as an adult	>3	3	8	50 %	89 %	27.3 %	0.77
				(3/6)	(64/72)	(3/11)	0.58–0.96
							0.031
*N* = 78	<3	3	64				
SIS and KIVS violence as an adult combined		5	14	83.3 %	80.3 %	26.3 %	0.85
				(5/6)	(57/71)	(5/19)	0.72–0.99
							0.009
*N* = 77		1	57				
KIVS exposure to violence as a child	>4	3	12	50 %	83.3 %	20 %	0.72
				(3/6)	(60/72)	(3/15)	0.53–0–90
							0.080
*N* = 78	<4	3	60				
SIS and KIVS exposure to violence as a child combined		6	30	100 %	58 %	17 %	0.77
				(6/6)	(41/71)	(6/36)	0.60–0.93
							0.04
*N* = 77		0	41				

#### Regression analysis

A standard logistic regression analysis was conducted with SIS and KIVS scores as predictors of suicide. The regression model was significant: chi square = 8.2, DF = 2, *p* = 0.017. Both SIS (chi square = 3.9, *p* = 0.049) and KIVS (chi square = 5.2, *p* = 0.022) ratings were statistically significant predictors of suicide in the regression model. After adding earlier repeated suicide attempt as a potential confounder, the model remained significant, chi square = 8.3, DF = 3, *p* = 0.041, with two significant predictors: SIS (chi square = 3.8, *p* < 0.050) and KIVS (chi square = 4.9, *p* = 0.026) scores, while the repeater status was not a significant predictor of subsequent suicide (chi square = 0.06, *p* = 0.80).

## Discussion

In this follow-up study of suicide attempters, we found that by combining the assessments for suicide intent and interpersonal violence in suicide attempters, we increased both the positive predictive value and the area under the curve. Combined use of both scales led to a higher specificity. Using both scales, the number of false-positives was reduced by nine patients compared to using SIS alone (26 vs. 35 false-positives) and by 17 patients compared to using KIVS alone (26 vs. 43 false-positives), leading to a specificity of 63 %. In other words, 11–21 % of suicide attempters in this cohort, classified as false-positive high suicide risk patients, could be reclassified as patients with a lower suicide risk after combining both scales in the suicide risk assessment. Using the optimal cut-offs of the rating scales, the suicide victims were clustered, having high ratings in both scales. In this study, the mean time to suicide after attempted suicide was 6 years. High scores on SIS or KIVS predicted suicide in the long term, indicating that the combined use may detect suicide attempters with a high risk of subsequent suicide from a long-term perspective. Interestingly, high scores on the KIVS subscale expressed interpersonal violence as an adult when combined with SIS gave a higher positive predictive value of 26.3 %, thereby improving the specificity while still showing quite a high sensitivity.

Theoretically, an optimal prediction model for suicide needs to have high sensitivity to detect all patients at risk for suicide and, if possible, also high specificity to reduce the number of false-positives; the latter being important due to the limited clinical resources [13]. Low specificity and a high number of false-positives have often been pointed out as a weakness when discussing the clinical utility of structured suicide risk assessment scales. A recent review pointed out that translating an elevated risk to the single individual falters because specific predictors are found among many individuals who are not suicidal (resulting in a high false-positive prediction) [5].

When assessing the suicide risk of self-harm patients, one must remember that results with low specificity are to be assumed in this population due to the wide range of the suicide intent and the actual degree of seriousness in the attempt. This is the reason why many clinical decision-making rules in, for example, emergency settings show low specificity in predicting repetition of self-harm in unselected self-harm populations [15]. Suicide is a rare outcome and is therefore, according to some researchers, impossible to predict with a degree of accuracy that is clinically meaningful [16, 17]. However, more well-designed follow-up studies focusing on the usefulness of structured suicide risk assessment scales in high-risk clinical groups are needed. A recent study identified the most discriminative items from a collection of scales usually employed in the assessment of suicidal behavior to compare suicide attempters with psychiatric inpatients without suicide attempts and healthy controls [18]. In this study, the participants were not followed up for repetition of attempted or completed suicide. In the future, the combined use of validated rating scales may be targeted on these groups, e.g., suicide attempters with high intent/violent methods and severe psychiatric disease, if studies with large clinical samples show further evidence of their predictive value.

Interestingly, there was a lack of significant positive correlation between the SIS and the KIVS, indicating that they are relatively independent and measure different components of suicide risk. Mann and coworkers have proposed that, given the multifactorial nature of suicidal behavior, a model that would incorporate several, largely independent, predictors would have greater predictive power [13]. The Beck SIS measures the degree of planning, lethal intent, preparation of the suicide attempt, communication, and precautions against intervention [7]. These aspects have been shown to be predictive of suicide in several studies [8]. The KIVS is a structured clinical instrument assessing exposure to violence and expressed violent behavior during the life cycle [12].

Childhood trauma has been shown to be a risk factor for suicidal behavior. Copeland et al. have recently concluded that the worst psychiatric effects of bullying can be seen in patients who were both victims and bullies [19]. The Adverse Childhood Experiences Study showed the strong relationship between adverse childhood experiences and the risk of attempted suicide [20]. Similarly, Klomek and colleagues conclude that suicides among girls could be reduced by 10 % if frequent victimization was eliminated [21]. Our results show the importance of using a structured interview, such as the KIVS, for retrieving information on previous victim and bully behavior in suicidal risk assessments. It is particularly important to detect the more vulnerable patients who have been exposed to violence in childhood and used violence as adults [12]. These two subscales of the KIVS had the highest predictive value for completed suicide [12] and showed an ability to distinguish patients at risk when combined with the SIS also in this study.

Impulsive and aggressive behaviors seem to underlie suicidal risk factors in major depression [22]. Suicide attempters had a higher risk of violent behavior than suicide ideators, making violent behavior one of the key differences between attempters and ideators [23]. In a long-term population based study of 167 Swedish homicide offenders followed up for 26 years, over 17 % of the homicide offenders committed suicide [24]. Furthermore, Lambert et al. observed in an American study of 473 participants that exposure to community violence was associated with increased aggressive behavior, which in turn was associated with suicide attempts [25]. Stenbacka et al. reported that violent offending and being victimized was associated with excess mortality and a risk of dying of suicide or of an alcohol or drug-related cause [26].

Both scales used in this clinical study have been studied in relation to underlying neurobiological vulnerability to suicidal behavior. Both high exposure to interpersonal violence as a child and accentuated aggression dyscontrol measured with the KIVS were associated with low levels of the serotonin metabolite 5-hydroxyindolacetic acid in the cerebrospinal fluid (CSF) of suicide attempters [27], whereas high suicide intent measured with the SIS was associated with low CSF oxytocin [28]. Furthermore, KIVS measures both the distal risk in the form of early life adversity and the developmental factor, impulsive aggression, while SIS may also capture more precipitating aspects of the suicidal crisis.

### Strengths and limitations

In this clinical high-risk group of suicide attempters followed up for between 15 and 20 years, seven patients committed suicide. Focusing on the high-risk patient group with a long follow-up time, we were able to study completed suicide as an outcome. The major strengths of this study are the simultaneous assessment of both the suicide intent and interpersonal violence, a careful diagnostic procedure, and the possibility of following up all patients in the nationwide registries for mortality. All causes of death were ascertained from the death certificates.

Some limitations should also be pointed out. We did not have structured information on nonsuicidal self-injury or which treatment or case management the patients received. Exact information on eligible patients during the study period was lacking. The small number of patients and completed suicides in this study is a limitation and conclusions should be taken with caution before replication in larger samples. Furthermore, there is a possibility of the change in predictivity being influenced by missing data since one of the suicide victims did not have a KIVS assessment at inclusion. The study was conducted in Sweden and there may be clinical traditions concerning which scales are used in different countries.

## Conclusion

We found that measuring both suicide intent and interpersonal violence together may be better for predicting suicide in suicide attempters with mood disorders and a high degree of comorbidity since the two instruments assess different aspects of the suicide risk. If replicated in large clinical studies, the results may influence clinical practice in the future.

## Additional file

Additional file 1:
**STROBE Statement—checklist of items that should be included in reports of observational studies.** (DOC 93 kb)

## References

[1] Tidemalm D, Långström N, Lichtenstein P, Runeson B (2008). Risk of suicide after suicide attempt according to coexisting psychiatric disorder: Swedish cohort study with long-term follow-up. BMJ.

[2] Runeson B, Tidemalm D, Dahlin M, Lichtenstein P, Långström N (2010). Method of attempted suicide as predictor of subsequent successful suicide: national long-term cohort study. BMJ.

[3] National Institute for Health and Care Excellence (2011). Self-harm (longer term management.

[4] Kendall T, Taylor C, Bhatti H, Chan M, Kapur N (2011). Longer-term management of self-harm: summary of NICE guidance. BMJ.

[5] Fowler JC (2012). Suicide risk assessment in clinical practice: pragmatic guidelines for imperfect assessments. Psychother.

[6] Posner K, Brown GK, Stanley B, Brent DA, Yershova KV, Oquendo MA (2011). The Columbia–Suicide Severity Rating Scale: initial validity and internal consistency findings from three multisite studies with adolescents and adults. Am J Psychiatry.

[7] Beck AT, Schuyler D, Herman I, Beck AT, Resnik HL, Lettieri DJ (1974). Development of suicidal intent scales. The Prediction of Suicide.

[8] Freedenthal S (2008). Assessing the wish to die: a 30-year review of the suicide intent scale. Arch Suicide Res.

[9] Stefansson J, Nordström P, Jokinen J (2012). Suicide Intent Scale in the prediction of suicide. J Affect Disord.

[10] O'Donnell O, House A, Waterman M (2015). The co-occurrence of aggression and self-harm: systematic literature review. J Affect Disord.

[11] Asellus P, Nordström P, Nordström AL, Jokinen J (2014). Cholesterol and the “Cycle of Violence” in attempted suicide. Psychiatry Res.

[12] Jokinen J, Forslund K, Ahnemark E, Gustavsson JP, Nordström P, Åsberg M (2010). Karolinska Interpersonal Violence Scale predicts suicide in suicide attempters. J Clin Psychiatry.

[13] Amsel L, Mann JJ, Appleby L (2001). Suicide risk assessment and the suicidal process approach. In Understanding Suicidal Behaviour: The Suicidal Process Approach to Research, Treatment and Prevention.

[14] Rajalin M, Hirvikoski T, Jokinen J (2013). Family history of suicide and exposure to interpersonal violence in childhood predict suicide in male suicide attempters. J Affect Disord.

[15] Bilén K, Ponzer S, Ottosson C, Castrén M, Pettersson H (2013). Deliberate self-harm patients in the emergency department: who will repeat and who will not? Validation and development of clinical decision rules. Emerg Med J.

[16] Large M, Sharma S, Cannon E, Ryan C, Nielssen O (2011). Risk factors for suicide within a year of discharge from psychiatric hospital: a systematic meta-analysis. Aust N Z J Psychiatry.

[17] Szmukler G (2012). Risk assessment for suicide and violence is of extremely limited value in general psychiatric practice. Aust N Z J Psychiatry.

[18] Blasco-Fontecilla H, Delgado-Gomez D, Ruiz-Hernandez D, Aguado D, Baca-Garcia E, Lopez-Castroman J (2012). Combining scales to assess suicide risk. J Psychiatr Res.

[19] Copeland WE, Wolke D, Angold A, Costello EJ (2013). Adult psychiatric outcomes of bullying and being bullied by peers in childhood and adolescence. JAMA Psychiatry.

[20] Dube SR, Anda RF, Felitti VJ, Chapman DP, Williamson DF, Giles WH (2001). Childhood abuse, household dysfunction, and the risk of attempted suicide throughout the life span: findings from the Adverse Childhood Experiences Study. JAMA.

[21] Klomek AB, Sourander A, Niemelä S, Kumpulainen K, Piha J, Tamminen T (2009). Childhood bullying behaviors as a risk for suicide attempts and completed suicides: a population-based birth cohort study. J Am Acad Child Adolesc Psychiatry.

[22] Dumais A, Lesage AD, Alda M, Rouleau G, Dumont M, Chawky N (2005). Risk factors for suicide completion in major depression: a case–control study of impulsive and aggressive behaviors in men. Am J Psychiatry.

[23] Stack S (2014). Differentiating suicide ideators from attempters: violence – a research note. Suicide Life-Threat Behav.

[24] Jokinen J, Forslund K, Nordström A-L, Lindqvist P, Nordström P (2009). Suicide risk after homicide in Sweden. Arch Suicide Res.

[25] Lambert SF, Copeland-Linder N, Ialongo NS (2008). Longitudinal associations between community violence exposure and suicidality. J Adolesc Health.

[26] Stenbacka M, Moberg T, Romelsjo A, Jokinen J (2012). Mortality and causes of death among violent offenders and victims - a Swedish population-based longitudinal study. BMC Pub Health.

[27] Moberg T, Nordström P, Forslund K, Kristiansson M, Åsberg M, Jokinen J (2011). CSF 5-HIAA and exposure to and expression of interpersonal violence in suicide attempters. J Affect Disord.

[28] Jokinen J, Chatzittofis A, Hellström C, Nordström P, Uvnäs-Moberg K, Åsberg M (2012). Low CSF oxytocin reflects high intent in suicide attempters. Psychoneuroendocrinology.

